# #StudentsToo. prevalence of sexual assault reports among students of three European universities and their actions post-assault

**DOI:** 10.1371/journal.pone.0283554

**Published:** 2023-04-07

**Authors:** Irena Boskovic, Robin Orthey, Henry Otgaar, Ivan Mangiulli, Eric Rassin

**Affiliations:** 1 Erasmus University Rotterdam, Rotterdam, The Netherlands; 2 Maastricht University, Maastricht, The Netherlands; 3 Kwansei Gakuin University, Nishinomiya, Japan; 4 KU Leuven, Leuven, Belgium; Caleb University, NIGERIA

## Abstract

**Objective:**

Previous research has indicated high rates of sexual assault (SA) among US students (> 25%). Yet this type of investigation has been less frequent at European universities.

**Methods:**

We conducted an investigation at three universities, two Dutch universities (*N* = 95 and *N* = 305) and one university in Belgium (*N* = 307). Students were asked to estimate the prevalence of SA, and to report about their personal experience. We defined SA as any situation in which students were inappropriately touched, forced to a sexual act without their consent, or were (sexually) verbally intimidated.

**Results:**

56% of students (Location 1: *n* = 54/95; Location 2: *n* = 172/305; Location 3: *n* = 172/307) across all three samples reported experiencing SA. The disclosed assaults were mostly unwanted sexual contact (i.e., groping) by male strangers aged 18–35 years. One-third of the sample reported to have done nothing post-assault, and among those who took actions, the majority disclosed the assault to friends, but rarely to family members. Also, 3–5% of students (Location 1: *n* = 3; Location 2: *n* = 11; Location 3: *n* = 11) (falsely) denied the assault. Seeking justice and needing support were important motivators of action, whereas psychological factors (i.e., memory distrust) were antagonists of this. Finally, besides psychological factors, fear of interpersonal consequences (e.g., being labelled as a “drama queen”) was a strong influence to either deny or try to forget the assault.

**Conclusion:**

SA appears to be frequent among European students and further investigation including other European universities is warranted.

## Introduction

Sexual assault (SA) is defined as any type of sexual contact or behaviour that occurs without the consent of the recipient [1]. It is estimated that up to 25% of women and 6% of men become victims of sexual assault [2]. A recent systematic review of SA research since 2010 showed that, specifically for Europe, the prevalence rates go from 0.3% to 56% (women: 6%-52%; men: 0.3%-56%; LGBT 7%-37% [3]). Yet, it is important to note that the SA prevalence often varies due to many different reasons, some of which are the varying definitions of sexual assault (e.g., including or not sexual harassment [4]) and the populations in which it is investigated.

Research revealed that the SA prevalence might be higher among college *students*, as they are among the most vulnerable groups to be sexually assaulted [5]. A survey involving 33 schools and universities in the US showed that 25.9% of undergraduate female students experienced non-consensual sexual contact by physical force or inability to consent (i.e., penetration and/or sexual touching), and 59.2% experienced sexual harassment [6]. A similar trend was observed also in the UK. A large-scale survey including more than 2000 female students showed that 68% of students reported being verbally or physically sexually harassed, whereas 14% reported severe SA (e.g., rape [7]). A more recent UK survey among female students indicated even higher numbers for sexual harassment (75%) and the same prevalence of severe SA (14% [8]). A study investigating a yearly prevalence of SA among Spanish female students showed that almost a third had experienced an assault during the previous 12 months (28.5% [9]). Regarding male students, the prevalence of SA is shown to be much greater than originally assumed (26% [10]), yet drastically lower than results including only female students. Indeed, in a recent Dutch national report on the prevalence of SA in Dutch students, this discrepancy between female and male students was obvious: Unwanted sexual act: 31% females, 11% male students; Unwanted sexual penetration: 18% female and 3% male students [11].

Besides gender, students’ sexuality also appears to be an important factor when investigating SA. Female bisexual students are the most likely to experience an assault, followed by students identifying as lesbian, and then heterosexual women [12]. Male students identifying as homosexual were more often victims of sexual assault than bisexual men, who were still more frequently assaulted than heterosexual men. Across all victim (gender and LGBTQAI+ (lesbian, gay, bisexual, transgender, queer, intersex, asexual, and more)) categories, the majority of perpetrators were male [13]. Interestingly, these findings are stable across countries, as almost identical results were observed in the UK reports [7, 8].

However, despite many estimates of SA prevalence, one of the major obstacles in accurately establishing the frequency of its occurrence is *underreporting*. According to the statistics of the Federal Bureau of Investigation (FBI), approximately 3 out of 4 SAs are unreported, making the perpetrators of sexual crimes less likely to go to prison than perpetrators of any other offense (National Crime Victimization Survey, NCVS) [14, 15]. Even more, underreporting is estimated to be more common among students than in the general population [16]. The results of UK survey indicated that most students (57%) often underestimate the severity of an assault, and therefore do not report it [8]. Students who are more likely to report an assault are those who seek justice, have trust in police, and understand the societal impact of reporting [1] (see also [17]). Yet the NCVS revealed that only 20% of college women who experienced a sexual assault filed a report to the police [18] (see also [14]). In the UK, the number of students who reported SA ranges between 14% and 21% [8], and these were mostly students who experienced a severe assault. Among students who were harassed, less than 2% reported the SA [7].

Currently, there is a rich body of literature investigating possible reasons for students to not report a SA including the characteristics of victims, perpetrators, and the situation in which SAs occur (see [1, 19–21]). For instance, Sable and colleagues [19] showed that the most important barriers for students to come forward after being assaulted were shame, guilt, embarrassment, or not wanting friends and family to know. Same results were found in the UK [7, 8] and among Dutch student samples [11]. Furthermore, students were also concerned about whether the report would be treated as confidential, whether they would not be believed, and how others would perceive them. Among female students, additional barriers emerged, such as fear of retaliation by the perpetrator, the perpetrator not allowing them to get help, protecting the perpetrator, and being financially dependent on the perpetrator [19]. Underreporting could also occur due to different reasons, such as victims’ low confidence in memory, or fear of appearing noncredible [20, 22]. Also, these factors might hinder a proper understanding of the situation. For instance, 60% of Dutch students with a history of being sexually penetrated against their will did not perceive that event as “rape”, due to either their intoxication, lack of violence, or other situational factors [11]. It must, however, be acknowledged that, very often, students do not actually know where to go and how to report an assault. In the survey among Dutch students, the majority of responders (64%), even if they wanted to, stated that they did not know where to go to report a SA. A similar percentage was also found in a large survey among medical students in Belgium (60%) [23].

Other, student-related, factors that significantly affect the likelihood of reporting an assault, are the characteristics of the situations in which it took place (see [20]). For instance, students are more likely to engage in consuming alcohol or drugs [24] (see also [25]). Further, more than half of the students who experienced mild to moderate sexual harassment were outside of campus, having a “night out”, and were harassed by strangers [7, 8]. Yet, the vast majority of *severely* assaulted female students already knew their perpetrator (80% [18]; 81% [7]). Both familiarity with the perpetrator and the consumption of alcohol were shown to decrease the likelihood of victims coming forward. Research has indeed shown that the cases in which a victim (i) refused to cooperate, (ii) consumed alcohol, (iii) knew the suspect or had a previous sexual relationship with the suspect, (iv) was uncertain about details, (v) or did not seem emotional during the report, were the most likely to be classified as *false* or unfounded [26]. However, these circumstances and behaviours were shown to be present also in SA cases with a substantial amount of evidence (e.g., [1, 27]). Hence, not being perceived as credible by police officers is a legitimate concern, and it is not surprising that approximately 70% of students chooses to rather disclose the assault to their (female) friends, a family member, intimate partners, psychologist or a social worker rather than to the police [7, 11, 28, 29] (see also [30]), and approximately 30% of students do not disclose the assault to anyone [8, 11].

It is additionally worrisome that police officers overestimate false allegations to be around 30% to 50% of reports, although their actual prevalence is around 2–6% [31]. Although false allegations, potentially leading to false positive outcomes of an investigation, are considered exceptionally worrisome, it is remarkable that false denials among adults, as an extreme end of underreporting which directly causes false negative conclusions, are, in comparison, seriously neglected among both researchers and practitioners.

### The current study

SA among students has been rigorously investigated mostly in the US, whereas Europe-based researchers have just recently started focusing on this topic. Due to the cultural, political, and social differences between the US and Europe, it is crucial to continue this line of investigation and properly establish the prevalence and the characteristics of SA among European students. Investigations as this could provide important information for further development and implementation of European university policies, risk education, and prevention programs. It is also important to note that this is the first project in which a prevalence of underreporting, by including reactions such as *doing nothing* and *falsely denying*, was also tested. Underreporting, specifically false denials, is often neglected, despite directly hindering the accuracy of previous findings regarding the SA prevalence.

Due to convenience, we chose to start this project with the universities we are affiliated with, however, the main purpose of this project is to launch further systematic, indepth and wider reaching investigation of SA among European students. We first conducted a small-scale survey among students of Maastricht university, the Netherlands (*Location 1*). The purpose of this survey was to establish a preliminary baseline and content validity of our questions. The next step in the project was to collect a larger sample of students from another university, specifically Erasmus University Rotterdam, the Netherlands (*Location 2*), in order to test the stability of the obtained prevalence. Finally, we conducted the same study at KU Leuven University, Belgium (*Location 3*), to examine the same factor in another country. We examined how often sexual assault occurs among students at these locations and the characteristics of such occurrences. Specifically, we asked the question about experiencing any form of SA (e.g., feeling unsafe, intimidation, verbal or physical harassment, severe assault), to which students could indicate either that they had direct experience, indirect experience (they know someone who was assaulted), or that they did not have any type of SA history. Further, we asked students to indicate their reactions post-assault and to write down the reasons they think influenced their choice of actions. As our project was explorative in nature, we did not make any predictions regarding our data. The data and the outputs are publicly available at Open Science Framework (https://osf.io/sfevc/; doi10.17605/OSF.IO/SFEVC).

To summarize, the main aims of this investigation were: 1. To establish the general prevalence of SA among Dutch and Belgian students. 2. To gain an insight into the main reactions (including both reporting and underreporting) students take post-SA, and 3. To understand what are the major influences behind the reactions of the assaulted youth.

## Method

### Participants

Our participants were recruited among psychology bachelor’s students at all three Universities. In Table 1, we present the descriptive characteristics of the samples used in all three locations (e.g., *N*, age, gender, sexual orientation etc.).

**Table 1:** Sample descriptives in all three study locations.

	*N*	*Age*	*Gender*	*LGBTQIA+*	*English proficiency* *	*Honesty* *
		*M* (*SD*)	% female	%	*M* (*SD*)	M (*SD*)
**Location 1**	95	19.88 (2.25)	87.4%	/	/	/
**Location 2**	305	20.62 (2.25)	82.6%	19%	4.32 (.74)	4.48 (1.13)
**Location 3**	307	19.24 (3.19)	85%	17%	3.90 (.71)	4.79 (.50)

*Notes*:

*Variables were measured using a 5-point Likert scale with lower values indicating lower levels.

Students at *Location 2* and *Location 3* were asked to grade the difficulty, discomfort, and clarity of our questions (5-point scale with low values indicating low levels). *Location 2* students reported low difficulty, *M* = 2.38, *SD* = 1.08, moderate discomfort, *M* = 2.52, *SD* = 1.15, and high clarity of questions, *M* = 4.43, *SD* = .88. *Location 3* participants also reported low difficulty, *M* = 2.60, *SD* = 1.02, moderate discomfort, *M* = 2.71, *SD* = 1.14, and high clarity of questions, *M* = 4.31, *SD* = .87.

### Measures

#### SA/Unsafe situation frequency, perpetrators’ characteristics

At *Location 1*, we administered questions pertaining to the frequency of sexual assault or the situations in which students felt unsafe, as well as about the familiarity of the perpetrator and perpetrator’s gender, and about the actions students took after the incident (for precise questions, see Supplemental material). At *Location 2*, we also included questions about participants’ sexual orientations and time of the assault. We also administered an open-ended question in which participants were asked to elaborate on the reasons behind their reactions to the unpleasant situations they experienced. These reasons were then coded (see *Coding the reasons*). The largest change from Location 1 was that participants who reported *not* having any negative experiences were then given the same questions but were asked to answer them as if they had had a SA experience. They were given the freedom to think of any hypothetical SA. Questions provided at the *Location 3* were the same as those at Location 2.

#### Reactions

Disclosing the assault to friends and to family is often the first step victims take post-assault [32], some victims, although shown as a minority, reported the assault to the police or people in charge (e.g., university counsellor), and some victims wanted to handle the situation themselves, hence, confront the assailant [1]. Yet, the majority of victims usually decided to do nothing [1, 19, 32, 33] (see also [34]). Therefore, we provided these potential reactions and left a possibility for students to add other reactions. It is important to emphasise that we also offered “denial” as an option, as prevalence rates of false denial of traumatic events such as sexual assualt are often debated, and mostly investigated only in children [35, 36]. Specifically, we offered the following reactions: Make a report to the police, Confronted the person, Tell my friends, Tell my family, Found a person in charge and filed a complaint, I did nothing, I denied that anything happened, Other (please insert). Also, at the *Location 2* and *Location 3* we added another potential reaction post-assault: trying not to think about it and forget it. This was decided due to further exploration of the literature which shows that some victims might actively try to forget about the assault [37, 38].

#### Coding the narratives

As we asked students at *Location 2* to also provide an elaboration about the reasons behind their choice of action post-SA, we coded all of the provided narratives. All narratives were coded by the first author and a research assistant who coded randomly determined 25% of the narratives. In order to make a list of the reasons students mentioned for the actions they took, we inspected their narratives and created groups of reasons. Because this investigation was exploratory and the reasons could not be predicted, we did not have any a priori categorization. The final list after reading all 305 narratives, included 45 different reasons (see S1 File for the original 45 reasons, their grouping, and coders’ agreement). We then examined all reasons and grouped them into 13 large themes of reasons (*Kappa*s > .84; see S1 File). The main 13 groups of reasons were: Lack of evidence (e.g., did not see the person), fear of personal consequences (e.g., retaliation), fear of interpersonal consequences (e.g., ruining friends dynamic; being seen as a “drama queen”), distrust in authorities (e.g., they would not do anything), situational factors (e.g., at the party/club, alcohol), social factors (e.g., religious parents, culture), psychological factors (e.g., memory distrust; delayed realization), seeking justice (e.g., that is wrong; I want to protect others), low severity assumption (e.g., “it wasn’t rape just groping”), emotional factors (e.g., shame, self-blame, shock), needing support and information (e.g., my friends would give me advice), giving the benefit of the doubt (e.g., he did not mean to do it), and presence of evidence (e.g., there were witnesses). Looking at data from *Location 3*, the first author again coded all narratives and a researcher assistant coded randomly determined 25% of the narratives. However, in this study, we coded the narratives directly according to the 13 themes of reasons defined in Study 2 (see S1 File for coders’ agreement; *Kappa*s > .80).

Further, from some of the narratives provided at Location 2 and Location 3, we could code the *severity of the assault* (*Kappa* = .90). We determined a priori that the information about the severity of the assault in participants’ narratives would be coded as follows: 0 –not explicitly mentioned; 1—verbal assault or intimidation (e.g., stalking); 2 –inappropriate touching by an unidentifiable person (e.g., groping in a club); 3 –inappropriate touching (groping) by an identifiable person; and 4 –severe assault (forced sexual contact including penetration; the victim was a minor). Looking at data from *Location 3*, the first author again coded all narratives and a researcher assistant coded randomly determined 25% of the narratives. However, in this study, we coded the narratives directly according to the 13 themes of reasons defined in Study 2 (see S1 File for coders’ agreement; *Kappa*s > .80). Further, from some of the narratives, as in Study 2, we could code the severity of the assault (*Kappa* = .88).

#### Procedure

Our questionnaires at all three locations were presented using the online platform, Qualtrics, in English. Informed consent was solicited first and only after students selected “Yes” they could continue with the study. Participants at Location 1 were administered a battery of various tests (unrelated to the current study) at the beginning of the academic year of 2019/2020). We included our list of questions in that battery. At Location 2, the data were collected from February until April 2020. Students joined the study using the university research participation system (SONA), where the study was posted. The data at Location 3 were collected during the period from Jun until October 2020. The study was also posted on the university research participation system (SONA), via which students could sign up for the study and receive the study link. All students were rewarded with research credits for their participation. All three studies received ethical approvals from the standing ethical committee of [Maastricht University, Erasmus University Rotterdam, and KU Leuven University]. Following the codes of ethics at these universities, all participants above 16 years of age were deemed sufficiently capable to participate and provide consent independently.

## Results

### Estimated frequency of sexual assault and unsafe situations among students

All of our participants were asked to rate how frequently students in general experience SA and unsafe situations on a scale that included the following options: “Never”, “Rarely”, “From time to Time”; “Often”, “Very often”, and “All the time”. In Table 2, we present the responses provided by students across the three locations. Overall, more than a third of students in all three samples (34%–44%) indicated that they are unsafe from time to time and a similar percentage reported being unsafe often (29%–38%).

**Table 2:** Participants’ estimations of SA frequency in student population in all three study locations.

	*N*	*How often do you think students are sexually assaulted or put in unsafe situations*?					
		Never	Rarely	From time to time	Often	Very often	All the time
**Location 1**	95	0	8%	38.9%	37.9%	14.7%	0
**Location 2**	305	0	16%	44%	29%	10%	0
**Location 3**	307	0.8%	8%	34.2%	38.4%	16.3%	2%

### The reported frequency of sexual assault and unsafe situations among students

In Table 3, we summarised the most relevant outcomes of our investigation. Across three locations, the numbers were almost identical, with 56% of students reporting direct SA experience, 15% indirect, and 28% having no SA history. Approximately, 65% of female participants and from 14 to 25% of male students experienced SA. Besides reporting about their SA experience, we also asked students how many times they have experienced SA or any similar unsafe situation. Students at *Location 1* indicated that, on average, it happened five times (*M* = 4.94, *SD* = 7.30; ranging from 1 until 50). Students at *Location 2* stated 7 occurrences (*M* = 7.42, *SD* = 11.08, range from 1 to 80), and students at *Location 3* reported, on average, 8 incidents (range from 1 to 100).

**Table 3:** Frequency (%) of students’ self-disclosed SA at Location 1, Location 2, and Location 3.

			Location 1	Location 2	Location 3
*N*			95	305	307
*Age M(SD)*			19.88 (2.25)	20.62 (2.25)	19.24 (3.19)
*SA History*					
**Direct**	Females	**Total**	67%	65%	63%
		Heterosexual		64.5%	60.4%
		LGBTQIA+	/	65.4%	75%
	Males	**Total**	25%	14%	17%
		Heterosexual		10.8%	12.5%
		LGBTQIA+		40%	34%
	LGBTQIA+	Total	/	64.5%	64%
	**Total %** (*n*)		**57%** (54)	**56%** (172)	**56%** (172)
**Indirect %** (*n*)			15% (14)	15% (47)	14% (44)
**Without%** (*n*)			28% (27)	28% (86)	29.5% (91)

*Note*: Percentages of Female, Male, and LGBTQIA+ are proportional to the size of the pertinent subsamples.

### Perpetrator’s characteristics

Students who reported either direct or indirect SA experience were asked to respond to a few questions regarding the perpetrator’s characteristics, such as gender, age, and relationship with the victim. Looking at the data from *Location 1*, for students who reported direct (*n* = 54) and indirect SA experience (*n* = 14), the perpetrator was male in the vast majority of cases (97%%; *n* = 66), and in 3% of cases it was a woman (*n* = 2). In 70% of reported cases, the perpetrator was a stranger (*n* = 48), whereas in 26.5% it was a friend of a victim (*n* = 18). In 10% of cases the perpetrator was a family member (*n* = 7). Participants also reported a perpetrator being an acquittance, boss, trainer/instructor, church counsellor, stepfather, roommate (20.5%; *n* = 14).

From *Location 2* students with direct and indirect SA history (*n* = 219), 95% (*n* = 207) reported that the perpetrator was male, whereas 4.5% reported that perpetrator was female (*n* = 10), and 1% did not know the gender. We also asked students about the age of the perpetrator, and 45% (*n* = 99) reported an age range from 18 until 25. Fourteen percent reported an age range 25–30 (*n* = 32), and 13% indicated that it was a minor (*n* = 28). For 61% (*n* = 132) of students the perpetrator was a stranger, whereas 13% reported that it was a friend (*n* = 29), 3% that it was a family member (*n* = 7), and 3.7% reported that it was a partner (*n* = 8). Fourteen percent added a different description (*n* = 30), such as classmate (3%), an acquaintance (2.5%), partners or family members (2%), teacher (1.4%), driving instructors (1%), guitar teachers (1%), and co-workers (1.5%). The reports about perpetrators’ characteristics among students of different sexual orientation are provided in the S1 File. We also asked students who never had an SA experience (*n* = 86) to tell us what they think the characteristics of the perpetrator in a hypothetical assault would be. Eighty percent thought that a perpetrator would be male (*n* = 68), and 26% (*n* = 24) thought the perpetrator’s age would be from 18 until 25, while the other 25% (*n* = 20) selected from 25 until 30. Further, 70% assumed that a perpetrator would be a stranger (*n* = 60), 8% that it would be a friend (*n* = 7), and 17% stated that it would be a familiar person, but not necessarily a friend (e.g., neighbour). Interestingly, only one person selected a family member option.

Finally, inspecting the *Location 3* data led to similar outcomes. Looking at the students with direct or indirect SA experience (*n* = 216), 94.5% reported that the perpetrator was male (*n* = 203), whereas 5.5% reported that the perpetrator was female. We also asked students about the age of the perpetrator. The majority (53.3%; *n* = 104) reported an age range from 18 until 25, 21.5% reported that the perpetrator was a minor (*n* = 42), 6.7% reported age range of 25–30 (*n* = 13), 2.6% the range 31–35 (*n* = 5), 4.6% reported the range of 36–40 (*n* = 9), and 11.3% of students disclosed that the perpetrator was older than 40 years (*n* = 22). When asked about the relationship with a perpetrator, 56.5% of students reported that it was a stranger (*n* = 118), whereas 18.7% reported that it was a friend (*n* = 39), 3% that it was a partner, 4.8% reported that it was a family member (*n* = 10), one person also reported it was a neighbour (0.5%). Sixteen per cent added a different description (*n* = 35), such as classmate (2.3%), acquaintance (5.6%), partners or friends of family members (1.5%), teachers/ instructors/ trainers (3%), and therapist (0.5%). The reports about perpetrators’ characteristics among students of different sexual orientation are provided in the S1 File. Students without SA history (*n* = 91) thought that a perpetrator would be male (93.7%, *n* = 74), and 53.6% thought the perpetrator’s age would be from 18 until 25 (*n* = 37), while 17.4% selected from 25 until 30 (*n* = 12). None of the participants selected a perpetrator could be a minor. Further, 86%% assumed that a perpetrator would be a stranger (*n* = 78), 6.6% that it would be a friend (*n* = 6), and 3.3% selected a family member (*n* = 3). Only one response was given for the remaining categories (partner and neighbour), and two participants added “acquaintance” as a response.

### Students’ reactions

The most frequently chosen reaction across three different study locations was “told my friends” (from 28% to 57%), “did nothing” (from 25% to 34%), and “confront the person” (from 14% to 21%; see Table 4). As participants who claimed no SA experience at *Location 2* and *Location 3* were then asked to imagine a hypothetical assault and to respond to the rest of the questions, we also present their responses in Table 4. Looking at the differences between the (in)direct experience and no experience group at both locations, students without SA history largely overestimated taking action (police report, report to person in charge) and telling family about the assault, and they also significantly underestimated doing nothing.

**Table 4:** Frequency of selected reactions at Location 1, Location 2 (among students with direct and indirect experience), and Location 3 (among students with direct and indirect experience).

Reactions	Location 1	Location 2					Location 3				
	% Group with (in)direct experience	% Group with (in)direct experience	% Group without experience	χ^2^(1)	*p*	*r*	% Group with (in)direct experience	% Group without experience	χ^2^(1)	*p*	*r*
*N*	60	219	86				216	91			
**Police report**	4%	3.2%	16.3%	16.48	**< .001**	.23	7.9%	38.5%	42.58	**< .001**	.37
Confronting the person	19%	14.2%	16.3%	.221	.638	.03	21.3%	18.7%	.27	.604	.03
Telling friends	31.6%	28.8%	27.9%	.022	.881	.008	56.9%	51.6%	.73	.394	.05
**Telling family**	17%	7.8%	23.3%	13.91	**< .001**	.21	13%	38.5%	25.52	**< .001**	.29
**Report to a person in charge**	3%	1.4%	9.3%	11.18	**.001**	.19	3.2%	15.4%	14.82	**< .001**	.22
**Do nothing**	25%	34.7%	3.5%	31.35	**< .001**	.32	28.2%	5.5%	19.63	**< .001**	.25
**Denial**	3%	5%	0%	4.48	**.034**	.12	5.1%	3.3%	.47	.491	.04
Trying to forget	/	0.5%	0%	.394	.530	.03	18.5%	24.2%	1.27	.259	.06
Something else (added responses)	12%	4.1%	3.5%	.06	.802	.01	9.7%	3.3%	3.67	.055	.11

*Notes*: Trying to forget was not included in Location 1 questionnaire.

### The time of sexual assaults and unsafe situations among students

At *Location 2* and *Location 3*, we had an additional question about the time of the assault. From *Location 2* students, who claimed to have had such an experience or know someone who did (*n* = 219), the majority reported that assaults occurred prior to their studies (55%; *n* = 120), whereas 27% (*n* = 59) reported that these situations occurred before and during their studies, and 18% (*n* = 39) of students reported that these experiences happened once they started their studies. The majority of *Location 3* students, who responded to have had such experiences or know someone who did, reported that SA occurred before their studies (65.3%, (*n* = 141), 18.5% reported that these situations occurred both before and during their studies (*n* = 40), while to 16.2% of students these experiences happened once they started their studies (*n* = 35).

### Severity of the assault

When conducting the study at *Location 2* and *Location 3*, we included a request for participants’ elaboration about the situation they experienced (i.e., students with direct SA history, (*Location 2*: *n* = 172; *Location 3*: *n* = 172). The majority of *Location 2* students did not explicitly mention the severity of the situation (59%, *n* = 101). From the rest of the narratives (*n* = 71), unwanted sexual contact by an unidentifiable person was the most commonly reported situation (e.g., groped in a club; 19.8%, *n* = 34), then being touched by a known person (e.g., touched or kissed by a friend or a friend of a friend;10%, *n* = 17), then being verbally intimidated and stalked (e.g., being followed home; 6.4%; *n* = 11), and situations which include severe assault (e.g., being 6 years old and being forced by a family member to kiss, or being 12 years old and being touched under the skirt by a stranger on a bus; 5.2%, *n* = 9).

Similar results were found among Location 3 students. Overall, the majority of students with direct SA exposure did not explicitly mention the severity of the situation (66%, *n* = 113). From the rest of the narratives, unwanted sexual contact by an unidentifiable person was the most commonly reported situation (17%, *n* = 29), then being touched by a known person (8.8%, *n* = 15), and situations which included severe assault (7%, *n* = 12; e.g., being raped, being sexually assaulted by a stepbrother, touched aggressively by a co-passenger on a plane, forcefully kissed/touched while being minor). Two accounts described verbal intimidation and stalking (1.2%).

### Reasons behind students’ reactions

Due to the narratives that we obtained at *Location 2* and *Location 3*, we looked into the reasons behind students’ reactions. We grouped the reasons into thirteen categories and checked how often were those reasons mentioned as influential factors for participants’ choice of action. For this analysis, we only selected students with either direct or indirect experience of an assault. *Location 2* students with (in)direct experience (*n* = 219) most frequently reported: Fear of personal consequences (20.5%, *n* = 45), distrust in authorities and others’ actions (19.2%, *n* = 42), low severity assumption (18.7%, *n* = 41), psychological factors (17.4%, *n* = 38), and situational factors (16%, *n* = 35). The seven other reasons were reported less frequently: Lack of evidence (13.7%, *n* = 30), emotional factors (11%, *n* = 24), seeking justice (9.6%, *n* = 21), fear of interpersonal consequences (8.7%, *n* = 19), social factors (3.7%, *n* = 8), needing support (3.2%, *n* = 7), giving a benefit of doubt (1.4%, *n* = 3), and present witnesses (1.8%, *n* = 4).

*Location 3* participants with either direct or indirect experience of an assault (*n* = 216) most frequently reported: Emotional factors (22.7%, *n* = 49), seeking justice (16.7%, *n* = 36), situational factors (16.2%, *n* = 35), needing support (15.7%, *n* = 34), and low severity assumption (14.8%, *n* = 32). The seven other reasons were reported less frequently: Fear of interpersonal consequences (13.4%, *n* = 29), psychological factors (11.6%, *n* = 25), fear of personal consequences (11.6%, *n* = 25), distrust in authorities and others’ actions (5.6%, *n* = 12), social factors (4.6%, *n* = 10), lack of evidence (3.7%, *n* = 8), present witnesses (2.3%, *n* = 5), and giving a benefit of doubt (1.9%, *n* = 4).

### Predicting students’ reactions

In order to explore whether we could predict students’ reactions post-assault based on the 13 groups of reasons they provided for their actions, we conducted binary logistic regressions including a combined sample of students with (direct) history of SA from *Location 2* (*n* = 172) and *Location 3* (*n* = 172). The sample started with 344 participants. Then, we excluded participants who indicated that they were not 100% honest during the survey (*n* = 53), leaving a sample of 291 participants for the calculation at step 1. Next, we focussed on those that did act to see what motivations drove the different actions, so we excluded all participants who indicated that they did nothing (*n* = 101). At this point, we also excluded participants who only indicated a course of action not described by our categories below. 15 participants were excluded for this reason, leaving a final sample of 175 for steps 2–5. We did not exclude any participants at these steps as multiple selections of reasons were possible. The exact test statistics can be found in Table 3, and the estimate indicates the log-odds in/decrease of performing an action when the corresponding reason is present. The same analyses were performed on the whole sample (*n* = 344), regardless of their honesty and are present in S1 File (Table 3). We computed an initial model that contained all 13 reasons as independent variables and the respective action variable as dependent variable. Then, we optimized the model with a stepwise AIC function, meaning that the model was penalized for the number of independent variables, so a better fit can be achieved by removing variables that are not predictive from the mode. We only report the final models.

First, we examined which reasons led victims to act at all (*step 1*). Victims with the desire to seek justice or need for support were more likely to take action (e.g., report, consult someone), while those who reported psychological factors were less likely to take action. For the following analyses, we excluded all students who did not take any action. Next, the action to seek help from the system (either police or superior at the place of the incident; *step 2*) was predicted by the need for support. Students who needed support were more likely to ask for help from the officials. However, seeking help from social environment (friends or family; *step 3*) was predicted mainly by distrust in authorities, needing support, perceiving the assault as less severe, and it was negatively predicted by seeking justice. Students who are more distrusting towards the police and officials, who need support, and who perceived the assault as less severe were more likely to ask for help from their environment. Yet students who seek justice were less likely to do so. The action to personally confront the offender (*step 4*) was predicted by one reason in a positive direction, namely seeking justice, and by two reasons in a negative direction: psychological factors (i.e., confusion, memory distrust) and low severity of the assault. Those who seek justice were more likely to confront the offender, while those who considered the assault not to be severe and who were confused, had delayed realization or distrust their memory of the event (i.e., psychological factors) were less likely to engage in a confrontation with the perpetrator. Finally, resorting to personal coping actions (not thinking about the incident or denying it happened; *step 5*) was predicted by students’ fear of interpersonal consequences following a SA and psychological factors (Table 5).

**Table 5:** Flowchart actions and reasons for self-reported victims of SA who reported being completely honest in their reports (Location 2 & Location 3; N = 291).

Steps	Estimate	*SE*	*z*	*p*
*Step 1*: *Did something*				
**Psychological factors**	**-0.69**	**0.33**	**-2.09**	**.037**
**Seeking justice**	**1.16**	**0.44**	**2.64**	**.008**
**Needing support**	**1.67**	**0.62**	**2.66**	**.008**
*Step 2*: *System help*				
**Needing support**	**1.90**	**0.58**	**3.25**	**< .001**
*Step 3*: *Social help*				
**Distrust in authorities**	**2.18**	**1.06**	**2.05**	**.040**
Situational factors	0.93	0.50	1.85	.064
**Seeking justice**	**-1.00**	**0.44**	**-2.24**	**.025**
**Low severity**	**1.38**	**0.66**	**2.09**	**.037**
**Needing support**	**2.44**	**0.78**	**3.14**	**.002**
Benefit of doubt	16.31	1385.37	0.01	.990
Presence of evidence	16.69	1340.71	0.01	.990
*Step 4*: *Personal–confrontation*				
**Psychological factors**	**-1.13**	**0.80**	**-1.41**	**.016**
**Seeking justice**	**2.23**	**0.44**	**5.07**	**< .001**
**Low severity**	**-1.70**	**0.81**	**-2.09**	.037*
*Step 5*: *Personal–coping*				
Lack of evidence	-17.66	2475.13	-0.007	.994
**Fear interpersonal consequences**	**1.67**	**0.71**	**2.33**	**.019**
Distrust in authorities	-18.21	2255.55	-0.008	.994
**Psychological factors**	**1.42**	**0.63**	**2.26**	**.024**
Seeking justice	-17.52	1765.54	-0.01	.992
Shame & self-blame	1.03	0.54	1.91	.056

*Notes*: Estimate indicates the in/decrease in log odds of that particular action if the reason is present. Reasons reported in bold were significant in the model.

## Discussion

Our results showed that the prevalence of self-disclosed sexual assault (SA) among European students is non-trivial. Students themselves rated SA frequency as relatively common. The assaults in half of the cases took place prior to starting the university. This information could suggest that half of the assaulted students were likely minors while experiencing (mostly) unwanted sexual contacts, and such incidents occurred approximately 5–8 times. This finding fits well with those previously discussed [1, 7] (see also [4, 19]). Further, students reported that the perpetrators of SA were usually male strangers, aged between 18–35 years old. The finding that the perpetrator was mostly a stranger seems to be specific to Europe, as the majority of US students report knowing the perpetrator [18]. It is possible that US students living on closed campuses might be more familiar with their peers than European students, although living on closed campus does not seem to increase the risk for SA [5, 39].

The prevalence of SA among European students we found, for both female and male, are very close to those reported in the previous investigations using US samples (60% for females [6], and 25% for males [10], respectively) and UK students [7, 8]. Yet our results indicate a higher SA prevalence than that reported in the recent Dutch report [11]. This discrepancy was probably caused by the different definitions of SA, as we also included situations in which students were made to feel (sexually) unsafe. Also, our sample consisted of all students (Dutch and international) attending the included universities, which might have had an impact on our findings. Further, because our SA prevalence is higher than the (yearly) one found among Spanish students [9], it is important to take into account the time period researchers were asking about in their investigations.

Notably, the SA prevalence is even higher when specifically looking at students who identify themselves as members of LGBTQIA+. However, it appears that females are more often victims of SA regardless of their sexuality, and the same outcome was found both in UK and US samples [7, 8, 12, 13] (see also [40]). Regarding men, those who identify themselves as LGBTQAI+ were approximately three times more likely to be assaulted than heterosexual men (see also [12, 13]). This finding is supported by previous research looking specifically into gay men and the abuse they face [41].

When examining the actions that students took post-assault, the similarities with US-based and UK-based research are also noticeable (see [7, 8, 18, 29]). Seeking peer support was the primary reaction, followed by confronting the person. Interestingly, doing nothing about the assault was in all three studies a more frequently exerted action than disclosing the assault to family members. Most students did not seek support and information primarily among their family, which might be due to the strong effect that disclosure could have not only on the single person, but on family dynamic as well. The reactions victims of SA receive from their environment can have serious effects on their future reporting behaviour. It was shown that the effect of positive reactions (e.g., support) is not as robust as the effect of negative reactions (i.e., judging, victim-blaming). A negative response from the environment was shown to be linked not only to future underreporting but also to victims’ depression, anxiety, and substance abuse problems [42].

It is crucial to also notice that approximately five percent of students denied the assault. This finding has not yet been reported in any of the available papers on the topic of SA among students. So far, research, especially in the domain of legal psychology, has focused on the prevalence of false allegations [43]. However, considering that the prevalence of false allegations is 2–6% [31, 44, 45], it is of utmost importance to inform the public that (false) denials of genuine victims arguably occur on a similar scale. Denial of an assault is an extreme end of the underreporting spectrum, which completely minimises the chances of prosecuting the perpetrator and preventing further assaults. Hence, more attention needs to be given to this issue in future SA research.

Unsurprisingly, students without SA history, when asked to imagine being assaulted and to select the post-assault reactions, significantly less frequently chose “do nothing” or for “deny the assault” option. However, taking official actions (e.g., police report and filing a complaint) and disclosing the assault to family members were overestimated by students without SA history. Still, these misestimations may be due to differences in the severity of imagined SA (e.g., rape) versus unwanted sexual contact (e.g., groping), which was the most commonly reported type of SA. Considering perpetrators’ characteristics, students without SA experiences were in line with responses of students with (in)direct SA history (i.e., male stranger, 18–35 years old). Overall, it seems that, without any type of reported SA experience, people are inaccurate in assuming the reactions one would take in such a situation, therefore, having an erroneous view that could support the rape myths and victim-blaming attitude (see also [1, 19, 27]).

### Prediction of students’ reactions

In our exploratory analyses, we investigated whether some of the reasons students at Location 2 and Location 3 with direct SA experience provided could predict their actions post-assault. Considering the situation as inappropriate and unjustifiable (seeking justice), as well as needing support were the main reasons encouraging the person to *do something* in those situations. This finding fits well with previous research also showing seeking justice as an important factor in coming forward after an assault (see [1]). However, psychological factors, such as memory distrust, delayed realization of the assault, and not understanding the severity of the situation at the moment were inhibiting taking an action (or driving the underreporting). Previous research showed that, among other characteristics, victims’ low confidence in their memory of the assault decreased the chances of them making a police report [32]. Further, needing support was also the driving influence for seeking system help or help in the environment (e.g., going to the person in charge, police, or telling friends). Yet it is noticeable that this reason was stronger in motivating students to disclose the assault to their environment, mostly to their peers, than going to the officials. Further, seeking help from the environment was also shown to be related to students’ distrust in authorities, and their perception of the assault as less severe. Students who find the situation inappropriate and unjustifiable, and tend to seek justice are less likely to resort to the environment’s support. Instead, they are more likely to confront the assailant. However, psychological factors and the perceived severity of the assault itself are also important factors. Students who were confused or had delayed realization of the assault were reasonably less likely to confront the person who assaulted them. Also, the less severe the assault is thought to be, the *less* likely it is for students to confront the person who assaulted them. This fits the results of the UK survey indicating that most victims of SA do not report it as they believe that what had happened to them was not “serious enough to report” [7, p. 4]. Finally, our data also indicated that reported psychological factors (e.g., memory distrust) and fear of interpersonal consequences, such as being seen as a “drama queen” or ruining friends’ dynamic, could predict students’ tendency to (*falsely*) *deny* the assault and to try not to think about it and hope to forget about the assault. This result confirmed (scarce) previous work on this topic. Namely, it was shown that denial of SA is most commonly evoked by social pressure and fear of social status damage (see [46]). Of course, the need to belong to a peer group is very strong among students, also seen through the engagement in hazing rituals. However, this finding suggests that students, due to their denial, perhaps remain in a group wherein the perpetrator is also part of, therefore, could be exposed to repeating victimization in future.

Overall, these findings support the literature addressed above showing that students are more vulnerable to SA [5], and are, at the same time, less likely to report such incidents [16]. Namely, seeing that students, as young adults, are fairly dependent on social groups, others’ acceptance, and lack confidence in systems that should protect them, it is not surprising that this population can fall prey under the pressure of maintaining “good” group dynamics and “reputation”. Hence, even if considering reporting the SA, students are prone to opt for reactions that will not lead to any legal impact nor consequences for the perpetrator. Therefore, it is important to continue this investigation and see how to lower SA victimization and encourage SA reporting among European youth.

### Limitations and implications

There are limitations to this work that warrant further consideration. First, the locations included in this project were of convenience, as the study was conducted at the universities we are affiliated with. However, this exploratory project was aimed to generate a wider investigation of SA among European universities, as it would be important to include more locations in the future steps of this research and evaluate the generalizability of our findings. Second, we used a very broad definition of SA, including also the instances of “feeling unsafe” due to the verbal intimidation, which is often neglected. We found this way of defining SA important, also from the educational perspective. As showed in the Dutch report, the majority of students consider only rape to be a sexual assault, and more than half of rape victims do not acknowledge the assault due to the lack of “typical” circumstances (e.g., violence). Further, a recent study has shown that victims often will not acknowledge the SA unless they are asked specific enough questions about the behaviour of the perpetrator [47]. However, the use of such a broad SA definition might have limited the clarity of the question for some participants. Third, and related to the previous point, we did not specifically instruct the “No SA” group about the type of SA they should imagine. It is likely that these students imagined the extreme cases of SA rather than mild or moderate ones (e.g., groping), which limits the comparison between the responses of assaulted and not assaulted students. Fourth, we did not track the nationality of our participants, thus, we could not investigate whether the prevalence of SA differs between international and Dutch/Belgian students. Based on some previous work, international students might be more vulnerable group than students are approximately closer to their support system [48]. Fifth, results regarding the age and the gender of the perpetrator might be simplified as students who indicated multiple SA experiences were not asked to specify these characteristics for each of the events, but rather to opt for one. Sixth, testing psychology students only, it is not surprising that we have gender inequality in our sample. Thus, our findings concerning male students might be of limited generalizability, although they fit well with the previously reported prevalence of SA specifically among male students [10, 19]. Finally, although we asked our participants to rate their honesty, we cannot guarantee that our students were completely truthful while responding to the questions. Specifically, we do not think our participants were deceiving, but as the topic is deeply uncomfortable, it is likely that our findings also reflect the issue of underreporting of SA. Hence, our results need to be taken with caution.

Taking these findings together, it is clear that education concerning SA among students and staff should be delivered via university policies (e.g., USVreact; see [49]), risk education, and prevention programs (see [50, 51]). Based on our data, the main issues that should be specifically stressed are 1) the prevalence of SA among students, its characteristics (e.g., that SA is far wider than rape), 2) typical reactions of the victims post-assault, and 3) counterbalancing factors which prevent students with SA history from taking actions, by, for instance, clearly informing students about the instances they should turn to (e.g., university counselor). Still, a necessary part of going forward within this line of research is directly asking European students about the ways in which they would want to be made safer while at the universities and taking their voices seriously. Our findings could serve as a guide for what aspects of SA need further elucidation and provide a framework for developing questions about psychological, situational, and motivational factors. It is necessary that the culture around SA changes in a way that the victims are not to be concerned about their reputation and potential scrutiny by others, as we found that especially those interpersonal concerns can impact victims to (falsely) deny an assault. However, it is also important to note that the majority of students were already assaulted prior to starting their studies, indicating that the topic of SA needs to be brought to the attention early in education.

## Conclusion

Overall, based on the three studies at two Dutch and one Belgian universities, it is evident that SA occurs in more than half of students. Students mostly reported unwanted sexual contact after which one third of assaulted students in all our samples did nothing, and only the minority took official actions such as filing a report (to the police or people/instances in charge). Also, it is important to note that 3–5% of students (falsely) denied the assault. The reasons behind the lack of action post-assault are mostly psychological in their nature (e.g., memory distrust, late realization of the assault, confusion), whereas seeking justice and support were the driving factors behind taking actions. The data from students without SA history showed inaccurate predictions of post-assault reactions victims usually take, which could contribute to “victim-blaming” culture.

## Supporting information

S1 File (DOCX)
