# Supplementary material for: #StudentsToo. prevalence of sexual assault reports among students of three European universities and their actions post-assault
Source: PLoS One. 2023 Apr 7;18(4):e0283554. doi: 10.1371/journal.pone.0283554 (PMC10081757; doi:10.1371/journal.pone.0283554)
Supplement: S1 File — (DOCX) [file pone.0283554.s001.docx]

**Supplemental File**

**Information letter for participants**

Dear students,

Thank you for your interest in our study!

In this project we wish to learn more about the frequency and characteristics of uncomfortable situations students might be exposed to, such as sexual assault. Sexual assault (SA) is defined as any type of sexual contact or behavior that occurs without the consent of the recipient. (e.g., unwanted physical contact or situations in which a person does not feel safe). You do not have to have history of such event in order to participate, as both students with and without such prior experiences are welcome.

We will present you with questions that tap into SA, which can be both general and personal. Before the questionnaire, you will be also administered demographic questions.

We are fully aware of the sensitivity of this topic, thus, at the end of the survey we will provide some contact information in case you, or someone close to you would like to reach out and talk about your experiences.

Your participation is completely anonymous.

The study will take (maximum) 20 minutes, and you will be awarded with 0.5 research credit for it. There are no foreseen risks of participating in this study, and, if you feel uncomfortable you can stop the participation in any given moment by exiting the study link.

If you need additional information, or you have questions, contact boskovic@essb.eur.nl

Thank you,

Irena Boskovic

**List of initial questions (Study 1)**

1. **How often do you think university students are sexually assaulted (e.g. inappropriately touched without their consent or verbally sexually assaulted)?**
   1. Rarely
   2. From time to time
   3. Often
   4. Very often
   5. All the time
2. **Has anyone ever touched your body inappropriately and without your consent or/and made you feel uncomfortable or not safe (verbally assaulted you)?** - Selected Choice
   1. Yes (please indicate how many time this has happened _________
   2. No
   3. Not me but it has happened to a person close to me
3. (if responses were a or c) **What was the gender of the perpetrator?**
   1. Male
   2. Female
   3. I don’t know
4. (if responses were a or c) **What was the age of the perpetrator?**
   1. Minor (<18)
   2. 18-25
   3. 26-30
   4. 31-35
   5. 36-40
   6. Above 40
   7. I don’t know
5. (if a or b) **Who was the perpetrator (click appropriate answers)?**
   1. A family member
   2. A friend
   3. A neighbor
   4. Other (please insert _________________________)
6. **What kind of action did you take, or the close person it happened to, after the incident? (click on appropriate answers)?**
   1. Make a report to the police
   2. Confronted the person
   3. Tell my friends
   4. Tell my family
   5. Found a person in charge and filed a complaint
   6. I did nothing
   7. I denied that anything happened
   8. I tried not to think about it and forget it
   9. Other (please insert ________________________)

**Added Questions (Study 2 and Study 3)**

*Demographic Questions*

**How would you define your sexuality?** (Please, respond to this question only if you feel comfortable doing so):

1. Heterosexual
2. Homosexual
3. Bisexual
4. Asexual
5. Prefer not to say
6. Other (please fill out)

*If the participants responded to Question 2 regarding the experience of SA with “Yes” or “Not me but it has happened to a person close to me”:*

**2b. Has this happened during your studies or while your close person was studying?**

1. Yes
2. No, prior to starting the uni
3. Prior to starting the uni and during my studies

**5b. Please, briefly elaborate on the reasons for opting for above presented action(s)** (e.g., were there any factors regarding the situation, you, or the other person that influenced this decision):

*If the participants responded to Question 2 with “No”:*

**2c. If something like that would happen to you, what gender *would you think* the perpetrator would be?**

**3. How familiar do you think that person would be to you? *Who do you think would be the most likely to assault you*?**

**4. What do you think *the age of the perpetrator would be*?**

**5. What kind of action *do you think you would take* after the incident (you can select multiple responses)?**

*Exit Questions*

**How honest do you think you were while responding to previous questions?** (Response format 5-point Likert scale)

**Interrater agreement Location 2**

| **Supplemental Table 1.**  *Interrater agreement on 45reasons and on thirteen categories of reasons (Location 2).* | | | |
| --- | --- | --- | --- |
| **13 Groups of Reason** | **45 Originally Coded Reasons** | *Kappa* (p< .001) | |
| **Severity of the assault** |  | .90 | |
| **Lack of evidence** | Not identifiable person | .78 | .90 |
|  | No evidence | 1.00 |  |
|  |  |  |  |
| **Fear of personal consequences** | Fear general | .79 | .91 |
|  | Fear of perpetrator action retaliation/perpetrator in position of power | .78 |  |
|  | Fear of consequences at school or work | / |  |
|  | Threat to say to others | / |  |
|  |  |  |  |
| **Fear of interpersonal consequences** | Fear of judgment stigma | 1.00 | 1.00 |
|  | Fear of reaction of my family | 1.00 |  |
|  | Fear of ruining family/friends dynamics | 1.00 |  |
|  |  |  |  |
| **Distrust in authorities** | I would not be believed | 1.00 | .92 |
|  | Distrust in authorities | 1.00 |  |
|  | Doing anything official would not do much/ it is pointless | 1.00 |  |
|  | Reporting it officially is too much hassle | / |  |
|  | I did not want to do anything | 1.00 |  |
|  | I wanted to handle it myself | 1.00 |  |
|  |  |  |  |
| **Situational factors** | Alcohol was involved | 1.00 | .90 |
|  | The perpetrator was a minor | / |  |
|  | I was a minor | .79 |  |
|  | It was my date | / |  |
|  | Not to make a scene | / |  |
|  | Lack of support in the environment | 1.00 |  |
|  |  |  |  |
| **Social factors** | Other victims did not report so neither did i | 1.00 | 1.00 |
|  | Culture normalization | 1.00 |  |
|  | Religion | 1.00 |  |
|  |  |  |  |
| **Psychological factors** | Memory distrust | 1.00 | .93 |
|  | Avoidance of thinking about it/ Fear of remembering/reexperience | .90 |  |
|  | Delayed realization of assault | 1.00 |  |
|  |  |  |  |
| **Seeking justice** | That is not okay/ Anger from crossing the boundaries | .79 | .90 |
|  | I wanted to embarass the perpetrator | / |  |
|  | I wanted punishment for perpetrator/ Justice | 1.00 |  |
|  | It physically harmed me so something needed to be done | / |  |
|  | To protect others | 1.00 |  |
|  | To protect my own safety and mental health | 1.00 |  |
|  |  |  |  |
| **Low severity assumption** | It is not severe enough | .81 | .84 |
|  | It was just one-time thing | 1.00 |  |
|  |  |  |  |
| **Emotional factors** | I put myself in that situation/ Selfblame | 1.00 | 1.00 |
|  | Disgust/ Shame/ Embaressment/ Guilt | .79 |  |
|  | Shock and surprise | 1.00 |  |
|  | Confusion about what to do | 1.00 |  |
|  |  |  |  |
| **Needing support and information** | Seeking support | .94 | 1.00 |
|  | Wanted to receive more information about what to do | .90 |  |
|  |  |  |  |
| **Giving the benefit of the doubt** | I wanted to protect the perpetrator | / | 1.00 |
|  | I was uncertain about his intent / Maybe it was not intentional | 1.00 |  |
|  |  |  |  |
| **Presence of evidence** | I had witnesses | 1.00 | 1.00 |
|  | I was not afraid / Presence of others | / |  |
| *Note:* As the second coder inspected 25% of the statements, some reasons were not found in selected narratives (“/”). | | | |

**Interrater agreement Location 3**

| **Supplemental Table 2.**  *Interrater agreement on 13 reasons (Location 3).* | | |
| --- | --- | --- |
| **Reasons** | ***Kappa*** | ***p*** |
| **Severity of the assault** | .88 | <.001 |
| **Lack of evidence** | 1.00 | <.001 |
| **Fear of personal consequences** | .82 | <.001 |
| **Fear of interpersonal consequences** | .87 | <.001 |
| **Distrust in authorities** | 1.00 | <.001 |
| **Situational factors** | .82 | <.001 |
| **Social factors** | 1.00 | <.001 |
| **Psychological factors** | .84 | <.001 |
| **Seeking justice** | .85 | <.001 |
| **Low severity assumption** | .85 | <.001 |
| **Emotional factors** | .80 | <.001 |
| **Needing support and information** | .84 | <.001 |
| **Giving the benefit of the doubt** | 1.00 | <.001 |
| **Presence of evidence** | 1.00 | <.001 |

**Perpetrator’s Characteristics Among Students Declaring as Heterosexual and LGBTQAI+**

**Location 2**

We also checked the reported perpetrator’s characteristics by straight and LGBTQAI+ participants. Considering the reports given by heterosexual participants, the perpetrator was male in 88.5% of cases (*n* = 218), female in 7% (*n* = 17), and of unknown gender in 4.5% (*n* = 11). Further, the age of the perpetrator was mostly between 18 and 25 (43%, *n* = 105), 25-30 range (17%, *n* = 41), or the perpetrator was a minor (9%, *n* = 22). Regarding the relationship with the perpetrator, it was a stranger in the majority of cases (65.5%, *n* = 159), or a friend (12.3%, *n* = 30), or something else (11%, *n* = 26). LGBTQAI+ participants reported being assaulted by male perpetrators (97%, *n* = 57), or the gender was unknown (3%, *n* = 2). The perpetrator was mostly 18-25 years of age (30.5%, *n* = 18), or older (25-30: 18.6%, *n* = 11; 31-35: 13.5%, *n* = 8). Also, in more than a half of reports, the perpetrator was a stranger (57%, *n* = 35), and less often a friend (10%, *n* = 6), or other (10%, *n* = 6).

**Location 3**

Here, we again checked the reported perpetrator’s characteristics by heterosexual and LGBTQAI+ participants. Heterosexual participants mostly reported that the perpetrator was male in 94.5% of cases (*n* = 225), and female in 5.5% (*n* = 13). Also, perpetrator’s age was mostly between 18 and 25 (46%, *n* = 114), then a minor (13%, *n* = 32), and an equal number of students marked age range 25-30 and above 40 (9%, *n* = 23). Regarding the relationship with the perpetrator, it was a stranger in the majority of cases (64%, *n* = 155), or a friend (14.5%, *n* = 65), or something else (12.5%, *n* = 30). Participants who declare as LGBTQAI+ also reported being assaulted by mostly male perpetrators (96.3%, *n* = 52), and female in 3.7% of cases (*n* = 2). The perpetrator was mostly 18-25 years of age (30.5%, *n* = 18), a minor (19%, *n* = 10), or older than 40 (15%, *n* = 8). Also, in more than a half of reports, the perpetrator was a stranger (55%, *n* = 32), and less often a friend (17%, *n* = 10), then family member (12%, n = 7), or other (12%, *n* = 7).

**Binary Regression Location 2 and Location 3**

Here are the results of the binary regression analyses on the combined sample from both Location 2 and Location 3 without exclusion based on the honesty question. The sample started with 344 participants. First, we excluded participants who did not indicate any of the possible actions. One participant was excluded for this reason, leaving a sample of 343 participants for the calculation at step 1.

After step 1 we focussed on those that did act to see what motivations drive the different actions, so we excluded all participants who indicated that they did nothing (reason 8). A total of 118 participants was excluded for this reason, leaving a final sample of 225 participants. At this point we also excluded participants who only indicated a course of action not described by our categories below. 15 participants were excluded for this reason, leaving a final sample of 210 for steps 2-5. We did not exclude any participants at these steps as multiple selections of reasons were possible.

| **Supplemental Table 3.**  *Flowchart actions and reasons for self-reported victims of SA.* | | | | | |
| --- | --- | --- | --- | --- | --- |
| Steps | | Estimate | *SE* | *z* | *p* |
| Step 1: Did something | |  |  |  |  |
|  | Distrust in authorities | -0.59 | 0.34 | -1.73 | .083 |
|  | **Psychological factors** | **-0.67** | **0.31** | **-2.17** | **.030*** |
|  | **Seeking justice** | **1.10** | **0.44** | **2.52** | **.011*** |
|  | **Needing support** | **1.63** | **0.62** | **2.60** | **.009*** |
|  | | | | | |
| Step 2: System help | |  |  |  |  |
|  | **Needing support** | **1.93** | **0.56** | **3.41** | **<.001*** |
|  | | | | | |
| Step 3: Social help | |  |  |  |  |
|  | Lack of evidence | 0.93 | 0.67 | 1.40 | .163 |
|  | Distrust in authorities | 1.45 | 0.77 | 1.88 | .060 |
|  | **Seeking justice** | **-0.85** | **0.42** | **-2.04** | **.041** |
|  | **Low severity** | **1.08** | **0.53** | **2.06** | **.039** |
|  | **Needing support** | **2.30** | **0.76** | **3.01** | **.003** |
|  | Benefit of doubt | 16.12 | 1385.38 | 0.01 | .991 |
|  | Presence of evidence | 16.58 | 1166.66 | 0.01 | .989 |
|  | | | | | |
| Step 4: Personal – confrontation | |  |  |  |  |
|  | Fear of personal consequences | -0.72 | 0.54 | -1.36 | .175 |
|  | **Seeking justice** | **2.18** | **0.41** | **5.19** | **<.001*** |
|  | **Low severity** | **-1.36** | **0.67** | **-2.04** | **.042*** |
|  | | | | | |
| Step 5: Personal – coping | |  |  |  |  |
|  | Lack of evidence | -16.85 | 1461.95 | -0.01 | .991 |
|  | Fear interpersonal consequences | 1.14 | 0.61 | 1.86 | .063 |
|  | Distrust in authorities | -1.76 | 1.09 | -1.61 | .108 |
|  | Social factors | 1.20 | 0.72 | 1.67 | .095 |
|  | **Psychological factors** | **1.29** | **0.53** | **2.43** | **.015** |
|  | Seeking justice | -1.85 | 1.05 | -1.75 | .080 |
|  | **Shame & self blame** | **0.90** | **0.46** | **1.97** | **.048** |
| *Notes*. Estimate indicates the in/decrease in log odds of that particular action if the reason is present. | | | | | |
